# Development and internal evaluation of an interpretable machine learning model based on clinical and radiomics features to differentiate lower extremity arterial embolism from atherothrombosis

**DOI:** 10.3389/fmed.2026.1827567

**Published:** 2026-08-10

**Authors:** Xiaodong Li, Hao Luo, Linzhuo Xie, Jian Shu, Xiaolei Sun

**Affiliations:** 1 Department of Interventional Medicine, The Affiliated Hospital of Southwest Medical University, Luzhou, China; 2 Department of Radiology, The Affiliated Hospital of Southwest Medical University, Luzhou, China; 3 The Fourth People’s Hospital of Sichuan Province, West China Chunxi Hospital of Sichuan University, Chengdu, China; 4 Department of General Surgery (Vascular Surgery), The Affiliated Hospital of Southwest Medical University, Luzhou, China; 5 Laboratory of Nucleic Acids in Medicine for National High-Level Talent, Nucleic Acid Medicine of Luzhou Key Laboratory, Southwest Medical University, Luzhou, China; 6 Key Laboratory of Medical Electrophysiology, Ministry of Education & Medical Electrophysiological Key Laboratory of Sichuan Province, Collaborative Innovation Center for Prevention and Treatment of Cardiovascular Disease of Sichuan Province, Institute of Cardiovascular Research, Southwest Medical University, Luzhou, China; 7 Cardiovascular and Metabolic Diseases Key Laboratory of Luzhou, Southwest Medical University, Luzhou, China; 8 The First Hospital of Liangshan, Xichang, China

**Keywords:** arterial embolism, atherothrombosis, diagnostic model, machine learning, radiomics

## Abstract

**Introduction:**

Accurate differentiation between lower extremity arterial embolism (AE) and atherothrombosis is essential. This study aimed to develop and validate an interpretable machine learning (ML) model combining clinical and radiomics features for preoperative non-invasive diagnosis.

**Methods:**

This retrospective study enrolled patients with lower extremity AE or atherothrombosis admitted to the Department of Vascular Surgery at our institution between January 2018 and January 2025. Clinical data were collected through the electronic medical record system, and radiomics features were extracted from lower extremity computed tomography angiography (CTA) images. Based on these features, three ML models were constructed, comprising a clinical model, a radiomics model, and a combined clinical-radiomics model. The diagnostic performance was evaluated using the area under the curve (AUC), sensitivity, specificity, F1-score, and accuracy. Furthermore, clinical utility was validated using calibration curves and decision curve analysis, while model interpretation was conducted via the SHAP method.

**Results:**

Among the three predictive models, the fusion model demonstrated the optimal differential diagnostic performance. In the testing set, this model yielded an AUC of 0.821 (95% CI 0.718–0.924), with sensitivity, specificity, F1-score, and accuracy of 0.750, 0.714, 0.761, and 0.734, respectively; the positive likelihood ratio (LR+) and negative likelihood ratio (LR-) were 2.62 and 0.35. In the training set, its AUC reached 0.979 (95% CI 0.959–0.999), with sensitivity, specificity, F1-score, and accuracy of 0.940, 0.939, 0.946, and 0.940, respectively; the LR+ and LR- were 15.41 and 0.06.

**Discussion:**

The clinical-radiomics fusion model demonstrates potential as a non-invasive auxiliary tool to assist in differentiating lower extremity atherothrombosis from AE in a single-center setting, warranting prospective multicenter validation.

## 1 Introduction

In the United States, the annual incidence of acute limb ischemia (ALI) is estimated to range from 16 to 25 cases per 100,000 population (1). Driven by the accelerating aging of the population, the overall incidence is increasing annually (2). Acute lower extremity ischemia is primarily caused by arterial embolism (AE) and atherothrombosis. Despite their similar clinical presentations, they differ fundamentally in thrombus origin and pathological composition (3). Consequently, therapeutic strategies diverge; for instance, atherothrombosis relies more on comprehensive medical management (including anticoagulation or thrombolysis) combined with revascularization strategies, whereas AE typically necessitates surgical intervention as the first-line treatment (4, 5). Thus, accurate etiological differentiation is paramount for guiding appropriate clinical management (5–8) and reducing the risks of amputation and mortality (9). Conventional diagnostic approaches rely heavily on subjective indicators, such as medical history and symptom characteristics (e.g., acuity of onset), rendering them prone to clinical misdiagnosis. However, a definitive diagnosis often requires surgical exploration or autopsy, which is typically unattainable for early clinical decision-making. Specifically, the emboli in AE are predominantly of cardiac origin (10) and are pathologically characterized as fibrin- and erythrocyte-rich ‘red thrombi’. In contrast, atherothrombosis is triggered by plaque rupture, initiating as a platelet-rich ‘white thrombus’ which may evolve into a mixed morphology (11, 12). However, visually distinguishing the subtle differences between these thrombus types remains challenging on computed tomography angiography (CTA) images (13). Radiomics, defined as a technique for extracting high-throughput quantitative features from medical images (14), enables the capture of patterns imperceptible to the naked eye. As a subset of artificial intelligence, machine learning (ML) excels in handling high-dimensional and non-linear data relationships, making it ideal for resolving complex classification problems that are intractable for conventional methods (15). While the ‘black-box’ nature of ML models has historically hindered clinical adoption, interpretability tools such as SHAP (Shapley Additive exPlanations) can significantly enhance prediction transparency and clinical trustworthiness by visualizing the underlying decision-making process (16).

The present study aims to develop and internally evaluate a preoperative, non-invasive diagnostic model utilizing interpretable ML based on lower extremity CTA radiomics features and clinical data, to differentiate between lower extremity AE and atherothrombosis in a single-center cohort.

## 2 Materials and methods

### 2.1 Patient selection

This retrospective study complied with the Declaration of Helsinki and was approved by the Ethics Committee of the Affiliated Hospital of Southwest Medical University (Approval No. KY2025163), which waived the requirement for informed consent. This study enrolled patients with lower extremity AE or atherothrombosis who underwent surgical treatment at our institution between January 2018 and January 2025. The inclusion criteria were: (1) patients who underwent surgical treatment for lower extremity thrombotic diseases at the Department of Vascular Surgery; and (2) patients with a confirmed postoperative diagnosis of AE or atherothrombosis. The exclusion criteria were as follows: (1) unavailability of preoperative lower extremity CTA images (*n* = 4); (2) excessive missing key clinical data (*n* = 10); (3) inability to perform reliable segmentation due to small lesion volume or severe artifacts (*n* = 24); (4) indeterminate etiology after intraoperative exploration, or thrombosis attributed to other specific etiologies (e.g., vasculitis or trauma) (*n* = 54); and (5) receipt of thrombolytic therapy or endovascular intervention prior to admission (*n* = 18). Following the aforementioned screening process, a total of 214 patients were enrolled in the study, comprising 94 cases of lower extremity AE and 120 cases of lower extremity atherothrombosis. The patient selection flowchart for this study is shown in Supplementary Figure S1.

### 2.2 Clinical data

The clinical data used in this study were extracted from the patients’ electronic medical records. Collected variables included demographic characteristics (sex and age), medical history (smoking, alcohol consumption, diabetes mellitus [DM], hypertension [HBP], coronary artery disease [CAD], atrial fibrillation [AF], and cerebrovascular disease [CeVD]), clinical symptoms (intermittent claudication [IC]), and the results of initial laboratory tests performed upon admission (encompassing complete blood count, liver and renal function, lipid profile, cardiac biomarkers, and coagulation function). In total, 65 variables were included in the analysis. During preliminary data organization, it was found that certain laboratory indices (such as N-terminal pro-B-type natriuretic peptide [NT-proBNP] and creatine kinase-MB [CK-MB]) contained extreme values beyond the instrument detection range. For these, results exceeding the upper detection limit were replaced with the upper limit value, while results falling below the lower detection limit were replaced with the value of the lower limit divided by√2. Missing values (primarily lipid profiles and cardiac biomarkers, <7%) were imputed using the K-nearest neighbors (KNN, K = 10) algorithm. To prevent data leakage, the replacement of out-of-range values and KNN imputation were performed using parameters derived solely from the training set and subsequently applied to the testing set.

### 2.3 CTA image acquisition, segmentation, and feature extraction

Lower extremity CTA was performed on a 256-slice helical scanner (Philips Healthcare) using the bolus tracking technique (triggered at the abdominal aorta). The scanning range extended from the third lumbar vertebra to the toes. Acquisition parameters were: tube voltage, 100 kV; tube current, 213 mA; matrix, 512 × 512; exposure time, 0.941 s; and slice thickness and interval, 0.9 mm. Images were retrieved from the Picture Archiving and Communication System (PACS) and imported into 3D Slicer (v5.6.1) for manual, slice-by-slice region of interest (ROI) segmentation on the axial plane. To ensure reproducibility, a random subset (n = 100) was re-segmented by a second blinded radiologist. The distribution of imputed values was consistent with the observed data, as shown in Supplementary Figures S3A–C.

After ROI delineation, radiomic features were extracted using FeAture Explorer (FAE, v0.5.13). The software is available on GitHub, and its functionalities are described in the developers’ publication (17). Image preprocessing included intensity normalization (scale: 1000), isotropic resampling (1 mm × 1 mm × 1 mm), and 25-bin Hounsfield unit discretization. The feature set comprised first-order, shape-based, and texture metrics. From 1,688 extracted features, 1,419 with an intraclass correlation coefficient (ICC) ≥ 0.80 were retained for subsequent modeling.

### 2.4 Feature processing and model construction

To optimize modeling performance, a stepwise preprocessing strategy was applied to all candidate variables. Initially, features with zero variance were excluded. Specifically, for radiomics features, degenerate features lacking clear biological significance (e.g., constant values or algorithmic placeholders) were removed to eliminate technical noise. Subsequently, continuous variables underwent winsorization, where values exceeding the 1st and 99th percentiles were capped. Spearman’s rank correlation analysis was then employed to assess inter-variable correlations; if the absolute correlation coefficient between any pair exceeded 0.90, one feature was discarded to mitigate multicollinearity. Finally, Z-score normalization was applied to all continuous variables. To prevent data leakage, winsorization, Spearman correlation-based filtering, and Z-score normalization were performed using parameters derived exclusively from the training set and subsequently applied to the testing set.

This study adopted a dual feature selection strategy combining least absolute shrinkage and selection operator (LASSO) regression and the Boruta algorithm. For LASSO, 10-fold cross-validation was employed to determine the optimal regularization parameter (*λ*), and features were selected based on the 1-standard-error (λ_1se_) rule. The Boruta algorithm, a Random Forest-based wrapper method, was utilized to evaluate variable importance, with tentative features resolved using a tentative rough fix strategy (18). Ultimately, the intersection of features identified by both methods was selected for modeling to enhance predictive stability. LASSO handles multicollinearity through L1 regularization and excels at identifying linearly associated predictors (19), whereas Boruta is a random-forest-based method capable of capturing non-linear relationships. The two methods are mechanistically complementary, and their intersection represents features that are consistently validated at both linear and non-linear levels, thereby reducing the risk of false positives and improving model generalizability (20).

In the construction of the clinical and radiomics models, nine ML algorithms were employed, covering linear classifiers (Logistic Regression, Naive Bayes), kernel-based methods (Support Vector Machine [SVM], Gaussian Process), ensemble learning (Random Forest, Gradient Boosting Machine [GBM], Extreme Gradient Boosting [XGBoost]), and neural network-based methods (Neural Network, Multilayer Perceptron [MLP]) (21, 22). For the fusion model, algorithms demonstrating superior performance in the clinical and radiomics models were selected. Hyperparameters for all models were optimized using 5-fold cross-validation.

### 2.5 Model evaluation

Model performance was assessed using the area under the curve (AUC), sensitivity, specificity, F1-score, and accuracy. Calibration was evaluated via calibration curves to assess the agreement between predicted probabilities and actual observed outcomes. Additionally, the Brier score was utilized to quantify the overall accuracy of probabilistic predictions (23). Clinical net benefit was analyzed using decision curve analysis (DCA). Additionally, positive and negative likelihood ratios (LR+ and LR-) were calculated to quantitatively estimate the clinical utility of the models. The optimal model was selected based on a weighted scoring system to balance overall discrimination and clinical utility. The formula was defined as: Weighted Score = 0.35 × AUC + 0.25 × Sensitivity + 0.20 × Specificity + 0.15 × F1-score + 0.05 × Accuracy. This weighting scheme prioritized AUC for overall performance, while assigning substantial weights to sensitivity and specificity to ensure clinical reliability.

### 2.6 Model explanation

Model interpretation was performed using the SHAP method. Rooted in cooperative game theory, SHAP utilizes Shapley values to assign a predictive contribution score to each feature. This approach allows for the visualization of variable importance proportions, thereby visually elucidating the decision-making process of the ML model (24).

### 2.7 Statistics

All statistical analyses were performed using R software (version 4.4.2). The normality of continuous variables was assessed using the Shapiro–Wilk test. Normally distributed data were expressed as mean ± standard deviation (SD) and compared using the independent samples t-test, while non-normally distributed data were presented as median (interquartile range [IQR]) and analyzed using the Mann–Whitney U test. Categorical variables were reported as counts (percentages) and compared using the Chi-square (χ^2^) test or Fisher’s exact test. All statistical tests were two-sided, and a *p*-value < 0.05 was considered statistically significant.

## 3 Results

### 3.1 Baseline characteristics and clinical model development

The entire study cohort was randomly partitioned into a training set (*n* = 150) and a testing set (*n* = 64) at a 7:3 ratio. Baseline characteristics are detailed in Table 1, while a comparison between the training and testing sets is provided in Supplementary Table S1. Analysis of the original data indicated that the atherothrombosis group was significantly older and predominantly male compared to the AE group (all *p* < 0.05). Furthermore, the atherothrombosis group exhibited significantly higher prevalence of IC, smoking, alcohol consumption, and HBP, whereas CAD and AF were more prevalent in the AE group (all *p* < 0.05). Laboratory analysis revealed that the atherothrombosis group had significantly higher platelet count (PLT), plateletcrit (PCT), eosinophil count and ratio (EOS and EOS-R), basophil count and ratio (BASO and BASO-R), and monocyte ratio (MONO-R), while neutrophil count and ratio (NEU and NEU-R) were significantly lower (all *p* < 0.05). Regarding coagulation profiles, the atherothrombosis group showed higher prothrombin time activity (PT-%), whereas prothrombin time (PT), prothrombin time-international normalized ratio (PT-INR), and prothrombin time ratio (PT-RATIO) were lower. Regarding liver function, the atherothrombosis group presented higher total bile acid levels (TBA), but lower gamma-glutamyl transferase (GGT), aspartate aminotransferase (AST), alanine aminotransferase (ALT), total bilirubin (TBIL), and direct bilirubin (DBIL). Notably, cardiac biomarkers including myoglobin (MYO), CK-MB, and high-sensitivity troponin T (hs-TNT) were significantly lower in the atherothrombosis group, along with lower NT-proBNP levels (all *p* < 0.01).

**Table 1:** Baseline characteristics by group.

Characteristic	Unit/levels	AE *N* = 94^a^	Atherothrombosis *N* = 120^a^	*P*-value^b^
Sex	-			<0.001
Female		45 (48%)	29 (24%)	
Male		49 (52%)	91 (76%)	
Age	Years	72.50 (60.00, 81.00)	78.00 (70.00, 84.00)	0.002
DM	-			0.228
No		75 (80%)	86 (72%)	
Yes		19 (20%)	34 (28%)	
HBP	-			0.016
No		58 (62%)	53 (44%)	
Yes		36 (38%)	67 (56%)	
CeVD	-			0.805
No		69 (73%)	91 (76%)	
Yes		25 (27%)	29 (24%)	
CAD	-			0.023
No		72 (77%)	107 (89%)	
Yes		22 (23%)	13 (11%)	
AF	-			<0.001
No		64 (68%)	109 (91%)	
Yes		30 (32%)	11 (9%)	
IC	-			<0.001
No		80 (85%)	56 (47%)	
Yes		14 (15%)	64 (53%)	
Smoking	-			<0.001
No		76 (81%)	67 (56%)	
Yes		18 (19%)	53 (44%)	
Alcohol	-			0.011
No		74 (79%)	74 (62%)	
Yes		20 (21%)	46 (38%)	
hs-TNT	μg/L	0.02 (0.01, 0.07)	0.02 (0.01, 0.02)	0.002
MYO	μg/L	153.60 (39.64, 1,112.00)	51.12 (29.32, 246.90)	0.001
CK-MB	μg/L	4.45 (1.97, 26.88)	2.66 (1.53, 6.57)	0.002
NT-proBNP	ng/L	1,604.68 (371.80, 3,571.00)	421.30 (189.00, 1,003.00)	<0.001
TC	mmol/L	4.51 (3.83, 5.28)	4.65 (3.91, 5.37)	0.437
TG	mmol/L	1.26 (0.97, 1.93)	1.31 (1.00, 2.05)	0.479
HDL-C	mmol/L	1.43 (1.11, 1.68)	1.22 (1.02, 1.60)	0.080
LDL-C	mmol/L	2.58 (1.94, 3.26)	2.85 (2.17, 3.26)	0.113
APOA1	g/L	1.43 (1.22, 1.66)	1.36 (1.17, 1.53)	0.084
APOB	g/L	0.88 (0.70, 1.06)	0.94 (0.81, 1.11)	0.139
WBC	×10^9^/L	10.27 (7.48, 12.94)	8.89 (6.73, 12.80)	0.085
NEU	×10^9^/L	8.45 (5.41, 11.82)	6.90 (4.77, 10.22)	0.036
LYM	×10^9^/L	1.12 (0.65, 1.59)	1.14 (0.72, 1.63)	0.604
MONO	×10^9^/L	0.55 (0.35, 0.74)	0.55 (0.41, 0.70)	0.699
EOS	×10^9^/L	0.01 (0.00, 0.07)	0.08 (0.02, 0.17)	<0.001
BASO	×10^9^/L	0.02 (0.01, 0.04)	0.03 (0.02, 0.04)	0.041
NEU-R	%	84.40 (74.60, 89.40)	78.35 (70.35, 85.75)	0.011
LYM-R	%	11.35 (5.80, 17.30)	12.85 (7.45, 20.15)	0.102
MONO-R	%	5.20 (3.30, 7.20)	6.25 (4.80, 7.65)	0.010
EOS-R	%	0.10 (0.00, 0.70)	0.90 (0.20, 2.20)	<0.001
BASO-R	%	0.20 (0.10, 0.40)	0.30 (0.20, 0.50)	0.005
RBC	×10^12^/L	4.38 ± 0.79	4.37 ± 0.73	0.940
HGB	g/L	135.00 (121.00, 150.00)	133.00 (120.00, 148.00)	0.466
HCT	-	0.41 (0.37, 0.45)	0.41 (0.37, 0.45)	0.642
MCV	fL	94.90 (90.80, 97.70)	94.25 (89.95, 97.85)	0.705
MCH	pg	31.00 (29.90, 32.30)	31.00 (29.25, 32.30)	0.762
MCHC	g/L	327.00 (321.00, 332.00)	327.00 (319.50, 333.00)	0.743
RDW-SD	fL	44.30 (42.70, 46.90)	44.95 (41.85, 48.30)	0.788
RDW-CV	%	13.55 (13.10, 14.50)	13.70 (13.05, 14.55)	0.510
PLT	×10^9^/L	177.50 (138.00, 239.00)	229.50 (152.00, 307.50)	0.005
MPV	fL	10.10 (9.00, 11.50)	9.65 (9.00, 10.70)	0.054
PCT	-	0.18 (0.15, 0.24)	0.22 (0.16, 0.29)	0.013
PDW	%	16.20 (15.90, 16.60)	16.20 (15.95, 16.50)	0.341
P-LCR	%	26.65 (20.50, 36.80)	24.60 (19.50, 31.45)	0.086
ALT	U/L	27.15 (16.50, 41.70)	18.55 (13.75, 32.90)	0.006
AST	U/L	32.10 (22.40, 59.00)	23.70 (18.25, 35.80)	<0.001
ALB	g/L	40.50 (37.20, 42.80)	39.55 (37.55, 42.80)	0.587
GLO	g/L	26.90 (24.70, 31.90)	28.45 (24.65, 32.75)	0.308
TBIL	μmol/L	13.60 (10.20, 19.30)	10.85 (7.45, 16.60)	0.031
DBIL	μmol/L	5.05 (3.30, 7.20)	3.90 (2.40, 6.80)	0.017
TBA	μmol/L	3.30 (2.00, 5.90)	4.40 (2.85, 7.20)	0.009
GGT	U/L	40.20 (25.10, 65.30)	31.80 (20.25, 53.15)	0.022
ALP	U/L	80.60 (66.00, 102.60)	78.75 (63.70, 102.75)	0.401
PA	mg/L	187.85 (150.50, 222.70)	182.35 (144.70, 234.95)	0.934
PT	s	13.60 (13.00, 14.50)	13.20 (12.50, 14.10)	0.014
PT-INR	INR	1.05 (0.98, 1.14)	1.01 (0.95, 1.09)	0.008
PT-RATIO	-	1.04 (0.98, 1.11)	1.01 (0.96, 1.07)	0.009
PT-%	%	92.00 (81.00, 103.00)	98.00 (87.50, 109.50)	0.011
APTT	s	33.35 (29.70, 39.10)	34.45 (30.25, 38.95)	0.422
TT	s	17.75 (16.50, 19.60)	17.50 (16.30, 18.40)	0.172
Fib	g/L	3.78 (3.02, 4.91)	3.95 (3.23, 5.59)	0.130
Urea	mmol/L	6.51 (5.35, 10.11)	6.51 (5.10, 8.72)	0.231
UA	μmol/L	339.20 (282.80, 438.30)	329.30 (241.45, 410.55)	0.051
Crea	μmol/L	74.80 (57.30, 96.80)	76.80 (62.40, 92.15)	0.893
GFR	mL/min	83.10 (53.10, 97.90)	82.50 (63.80, 90.85)	0.807

AE, arterial embolism; AF, atrial fibrillation; ALB, albumin; ALP, alkaline phosphatase; ALT, alanine aminotransferase; APOA1, apolipoprotein A1; APOB, apolipoprotein B; APTT, activated partial thromboplastin time; AST, aspartate aminotransferase; BASO, basophil count; BASO-R, basophil ratio; CAD, coronary artery disease; CeVD, cerebrovascular disease; CK-MB, creatine kinase-MB; Crea, creatinine; DBIL, direct bilirubin; DM, diabetes mellitus; EOS, eosinophil count; EOS-R, eosinophil ratio; Fib, fibrinogen; GFR, glomerular filtration rate; GGT, gamma-glutamyl transferase; GLO, globulin; HBP, hypertension; HCT, hematocrit; HDL-C, high-density lipoprotein cholesterol; HGB, hemoglobin; hs-TNT, high-sensitivity troponin T; IC, intermittent claudication; LDL-C, low-density lipoprotein cholesterol; LYM, lymphocyte count; LYM-R, lymphocyte ratio; MCH, mean corpuscular hemoglobin; MCHC, mean corpuscular hemoglobin concentration; MCV, mean corpuscular volume; MONO, monocyte count; MONO-R, monocyte ratio; MPV, mean platelet volume; MYO, myoglobin; NEU, neutrophil count; NEU-R, neutrophil ratio; NT-proBNP, N-terminal pro-B-type natriuretic peptide; P-LCR, platelet-large cell ratio; PA, prealbumin; PCT, plateletcrit; PDW, platelet distribution width; PLT, platelet count; PT, prothrombin time; PT-%, prothrombin time activity; PT-INR, prothrombin time-international normalized ratio; PT-RATIO, prothrombin time ratio; RBC, red blood cell; RDW-CV, red cell distribution width-coefficient of variation; RDW-SD, red cell distribution width-standard deviation; TBA, total bile acid; TBIL, total bilirubin; TC, total cholesterol; TG, triglyceride; TT, thrombin time; UA, uric acid; WBC, white blood cell.

a n (%); Median (Q1, Q3); Mean±SD.

b Continuous variables: normally distributed data are presented as mean ± SD and compared using Welch *t*-test; non-normally distributed data are presented as median (IQR) and compared using Mann–Whitney U test. Categorical variables are presented as *n* (%) and compared using chi-square test. All *p*-values are rounded to three decimal places.

To establish a simple clinical baseline reflecting routine clinical assessment, a basic logistic regression model was constructed using clinically meaningful variables for differential diagnosis (IC and AF) along with age. This simple baseline model achieved a training AUC of 0.812 and a testing AUC of 0.703 (Supplementary Figure S2), serving as a fundamental reference for evaluating the incremental value of subsequent complex models.

**Figure 1:**
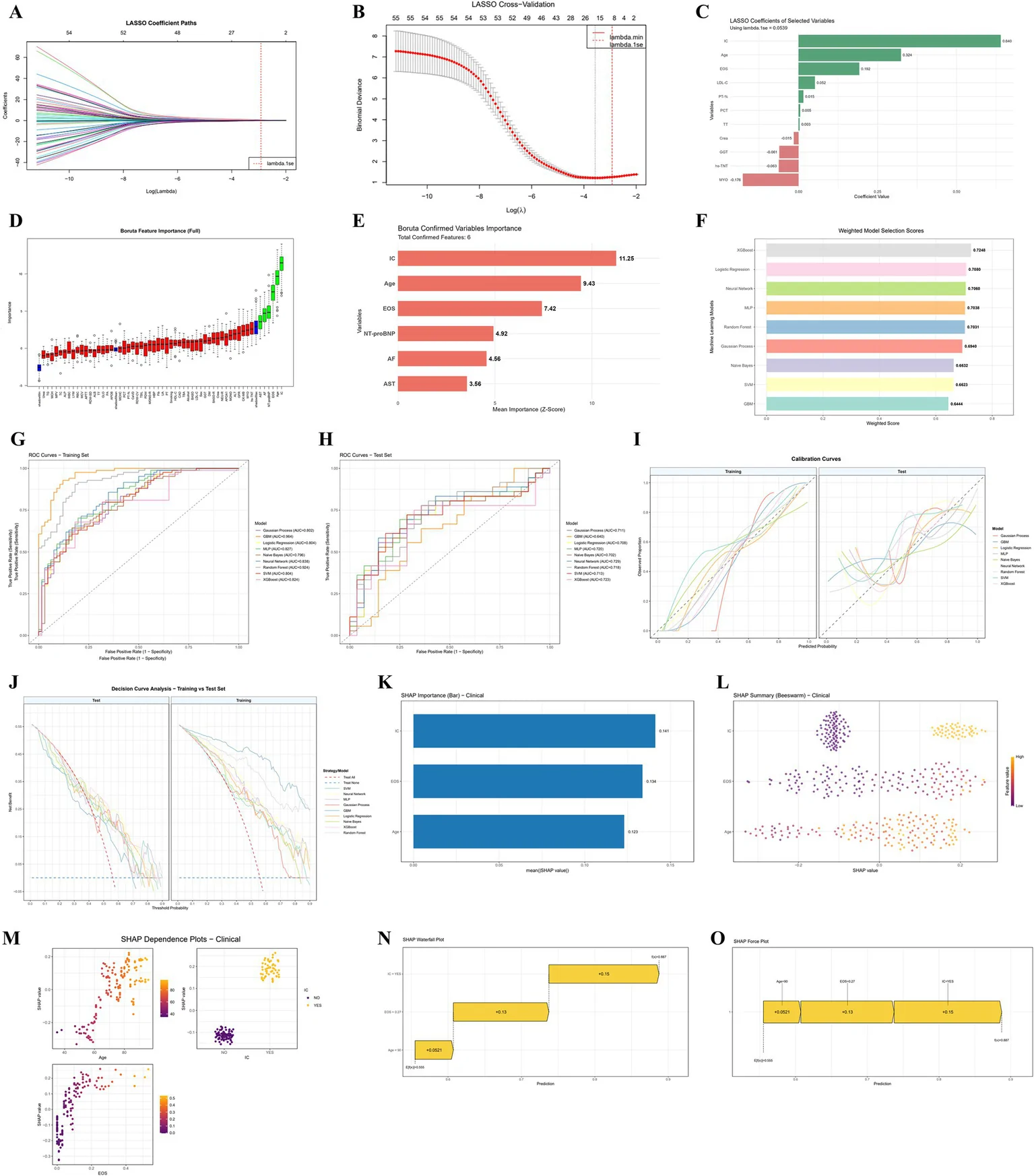
Variable selection, performance evaluation, and interpretability analysis of the clinical ML model. **(A)** LASSO coefficient path plot of clinical features. **(B)** Ten-fold cross-validation curve of LASSO regression. **(C)** Coefficient plot of clinical variables selected by LASSO. **(D)** Boruta feature importance boxplots. **(E)** Importance ranking plot of clinical variables by the Boruta algorithm. **(F)** Horizontal bar chart of the weighted performance score ranking for ML algorithms in the test set. **(G)** ROC curves of ML models in the training set. **(H)** ROC curves of ML models in the test set. **(I)** Calibration curves of the clinical model in the training and test sets. **(J)** Decision curve analysis of the clinical model in the training and test sets. **(K)** SHAP global importance bar plot of the clinical model. **(L)** SHAP summary beeswarm plot of the clinical model. **(M)** SHAP dependence plots of clinical variables. **(N)** SHAP waterfall plot for a typical high-risk sample (Sample 10). **(O)** SHAP force plot for a typical high-risk sample (Sample 10). LASSO, least absolute shrinkage and selection operator; ML, machine learning; ROC, receiver operating characteristic; SHAP, SHapley Additive exPlanations.

After excluding collinear variables (Supplementary Figure S3D), a total of 55 clinical variables were retained for subsequent analysis. LASSO regression identified 11 variables with non-zero coefficients, including IC, age, EOS, MYO, hs-TNT, GGT, low-density lipoprotein cholesterol (LDL-C), PT-%, creatinine (Crea), PCT, and thrombin time (TT) (Figures 1A–C), while the Boruta algorithm selected 6 key variables, comprising IC, age, EOS, NT-proBNP, AF, and AST (Figures 1D,E). Based on the intersection of variables selected by the two methods, IC, age, and EOS were ultimately identified as predictors and utilized for subsequent model construction. Based on the three aforementioned variables, modeling results indicated that among the nine ML algorithms, XGBoost was selected as the optimal model, achieving a weighted score of 0.725 (Figure 1F). In the training set, this model yielded an AUC of 0.824 (95% confidence interval [CI]: 0.760–0.888) (Figure 1G), with a sensitivity of 0.798, specificity of 0.682, F1-score of 0.779, and accuracy of 0.747 (Table 2). In the testing set, the AUC was 0.723 (95% CI: 0.598–0.848) (Figure 1H), while sensitivity, specificity, F1-score, and accuracy were 0.750, 0.679, 0.750, and 0.719, respectively. Additionally, the LR + and LR- were 2.34 and 0.37, respectively (Table 2). Calibration curve analysis revealed that the XGBoost model demonstrated good consistency in both training and testing sets, exhibiting the best calibration performance among all models (Figure 1I). Specifically, in the testing set, XGBoost achieved the lowest Brier score (0.208) and the most ideal calibration slope (1.159) (Supplementary Table S2). DCA curves indicated that XGBoost provided a positive net benefit across a broad threshold probability range, suggesting its practical utility across most clinical decision thresholds (Figure 1J).

**Table 2:** Performance metrics of the clinical prediction model on the training and test sets.

Model	Dataset	AUC (95% CI)	Accuracy	Sensitivity	Specificity	F1	LR+	LR–
SVM	Training	0.804 (0.735–0.873)	0.7	0.595	0.833	0.69	3.56	0.49
SVM	Test	0.713 (0.583–0.842)	0.641	0.5	0.821	0.61	2.79	0.61
Neural network	Training	0.838 (0.774–0.901)	0.753	0.833	0.652	0.791	2.39	0.26
Neural network	Test	0.729 (0.601–0.857)	0.688	0.694	0.679	0.714	2.16	0.45
MLP	Training	0.827 (0.762–0.892)	0.733	0.726	0.742	0.753	2.81	0.37
MLP	Test	0.72 (0.591–0.848)	0.688	0.722	0.643	0.722	2.02	0.43
Gaussian process	Training	0.802 (0.733–0.872)	0.72	0.845	0.561	0.772	1.92	0.28
Gaussian process	Test	0.711 (0.580–0.842)	0.672	0.806	0.5	0.734	1.61	0.39
GBM	Training	0.964 (0.939–0.989)	0.9	0.964	0.818	0.915	5.30	0.04
GBM	Test	0.64 (0.500–0.781)	0.641	0.639	0.643	0.667	1.79	0.56
Logistic regression	Training	0.804 (0.734–0.873)	0.707	0.726	0.682	0.735	2.28	0.40
Logistic regression	Test	0.708 (0.576–0.839)	0.703	0.694	0.714	0.725	2.43	0.43
Naive Bayes	Training	0.796 (0.725–0.867)	0.713	0.643	0.803	0.715	3.26	0.44
Naive Bayes	Test	0.702 (0.569–0.835)	0.641	0.583	0.714	0.646	2.04	0.58
XGBoost	Training	0.824 (0.760–0.888)	0.747	0.798	0.682	0.779	2.51	0.30
XGBoost	Test	0.723 (0.598–0.848)	0.719	0.75	0.679	0.75	2.34	0.37
Random forest	Training	0.924 (0.884–0.964)	0.833	0.833	0.833	0.848	4.99	0.20
Random forest	Test	0.718 (0.590–0.845)	0.688	0.722	0.643	0.722	2.02	0.43

AUC, area under the curve; CI, confidence interval; GBM, gradient boosting machine; MLP, multilayer perceptron; SVM, support vector machine; XGBoost, extreme gradient boosting; LR+, positive likelihood ratio; LR–, negative likelihood ratio.

AUC quantifies the overall discriminative ability across all thresholds. Accuracy is the proportion of all correct predictions. Sensitivity measures the proportion of actual positives that are correctly identified. Specificity measures the proportion of actual negatives that are correctly identified. The F1-score represents the harmonic mean of precision and sensitivity, providing a balanced metric especially for imbalanced datasets.

To provide a global interpretation of the prediction mechanism of the clinical model (XGBoost), SHAP feature importance bar plots, beeswarm plots, and dependence plots were generated. The SHAP importance bar plot revealed that among the three incorporated clinical features, IC made the greatest contribution to model prediction, followed by EOS and age (Figure 1K). Furthermore, the SHAP beeswarm (Figure 1L) and dependence plots (Figure 1M) demonstrated that the presence of IC, elevated EOS levels, and advanced age were all associated with an increased risk of atherothrombosis as predicted by the model. To further facilitate local model interpretation, a single-sample analysis was performed on a high-risk patient (Sample 10), for whom the predicted probability of atherothrombosis was approximately 0.887. SHAP force plots (Figure 1N) and waterfall plots (Figure 1O) quantified the independent contributions of specific feature values to the prediction. Notably, the presence of IC made the largest contribution (SHAP value = +0.15), followed by elevated EOS levels (+0.13) and advanced age (+0.051).

### 3.2 Radiomics model development

**Figure 2:**
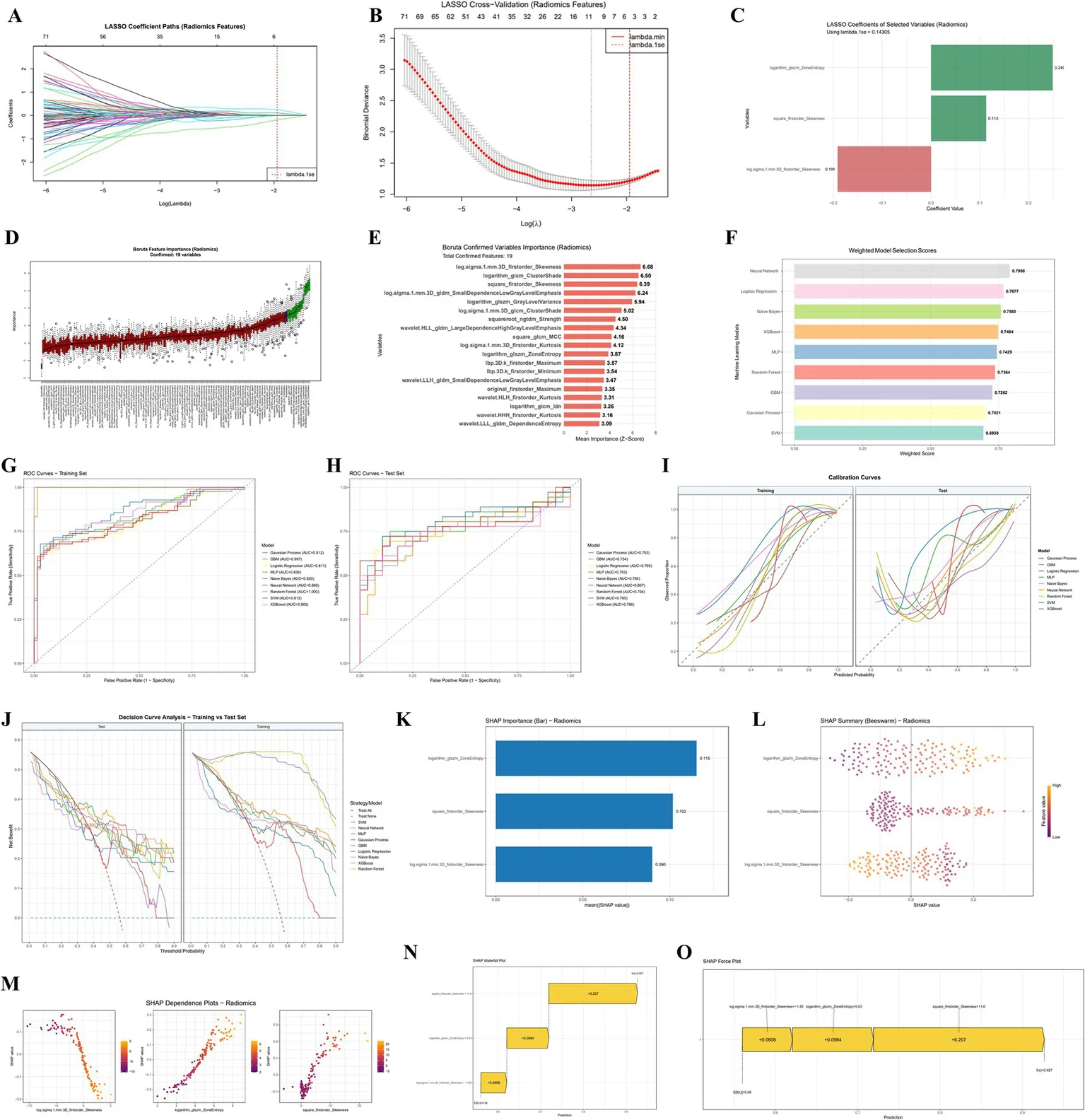
Variable selection, performance evaluation, and interpretability analysis of the radiomics ML model. **(A)** LASSO coefficient path plot of radiomics features. **(B)** Ten-fold cross-validation curve of LASSO regression. **(C)** Coefficient plot of radiomics variables selected by LASSO. **(D)** Boruta feature importance boxplots. **(E)** Importance ranking plot of radiomics variables by the Boruta algorithm. **(F)** Horizontal bar chart of the weighted performance score ranking for ML algorithms in the test set. **(G)** ROC curves of ML models in the training set. **(H)** ROC curves of ML models in the test set. **(I)** Calibration curves of the radiomics model in the training and test sets. **(J)** Decision curve analysis of the radiomics model in the training and test sets. **(K)** SHAP global importance bar plot of the radiomics model. **(L)** SHAP summary beeswarm plot of the radiomics model. **(M)** SHAP dependence plots of radiomics variables. **(N)** SHAP waterfall plot for a typical high-risk sample (Sample 10). **(O)** SHAP force plot for a typical high-risk sample (Sample 10). LASSO, least absolute shrinkage and selection operator; ML, machine learning; ROC, receiver operating characteristic; SHAP, SHapley Additive exPlanations.

After feature preprocessing, 219 candidate radiomics features were retained for subsequent analysis. Among these, LASSO regression with 10-fold cross-validation selected 3 features (Figures 2A–C). The Boruta algorithm selected 19 features, encompassing first-order statistics, multiple texture features—including gray-level co-occurrence matrix (GLCM), gray-level size zone matrix (GLSZM), gray-level dependence matrix (GLDM), and neighboring gray tone difference matrix (NGTDM)—and high-order features derived from wavelet, logarithmic filtering, and local binary pattern (LBP) operators (Figures 2D,E). Based on the intersection of the two feature selection methods, three features were ultimately identified for the construction of the radiomics model: log.sigma.1.mm.3D_firstorder_Skewness, logarithm_glszm_ZoneEntropy, and square_firstorder_Skewness. Based on the 100 cases with repeat segmentation, the ICC values of these three features were 0.905, 0.971, and 0.819, respectively (Supplementary Table S3), confirming high inter-observer reproducibility. To evaluate the selection stability of these three features, bootstrap resampling (1,000 iterations) was performed on the training set. The feature log.sigma.1.mm.3D_firstorder_Skewness was selected in 92.2% of bootstrap samples, while logarithm_glszm_ZoneEntropy and square_firstorder_Skewness showed selection frequencies of 54.4 and 46.5%, respectively (Supplementary Table S4). While the latter two features exhibited moderate selection frequencies—reflecting the inherent instability of aggressive feature reduction in high-dimensional, small-sample settings—they were retained because they represented the unique consensus intersection of both LASSO and Boruta algorithms on the full training set, and were therefore used for subsequent modeling. Nine ML models were constructed using the intersected radiomics features. The results indicated that the Neural Network performed best in the comprehensive score (0.791) and was identified as the optimal radiomics model (Figure 2F). In the training set, the Neural Network yielded an AUC of 0.866 (95% CI 0.809–0.922) (Figure 2G), a sensitivity of 0.679, a specificity of 0.924, an F1-score of 0.781, and an accuracy of 0.787 (Table 3). In the testing set, the AUC was 0.807 (95% CI 0.695–0.918) (Figure 2H), and the sensitivity, specificity, F1-score, and accuracy were 0.694, 0.893, 0.781, and 0.781, respectively. Additionally, the LR + and LR- were 6.49 and 0.34, respectively (Table 3). Compared with the previously constructed optimal clinical model (XGBoost), the radiomics model (Neural Network) exhibited higher specificity (0.893 vs. 0.679) and discriminative ability (AUC 0.807 vs. 0.723) in the testing set, whereas the clinical model demonstrated higher sensitivity (0.750 vs. 0.694). Calibration analysis indicated that among all ML models (Figure 2I), the Neural Network achieved the lowest Brier score (0.178) in the testing set (Supplementary Table S5), suggesting relatively superior predictive accuracy. Furthermore, DCA revealed that the Neural Network provided a positive net benefit across a broad range of threshold probabilities, outperforming the treat-all and treat-none strategies (Figure 2J).

**Table 3:** Performance metrics of the radiomics model on the training and test sets.

Model	Dataset	AUC (95% CI)	Accuracy	Sensitivity	Specificity	F1	LR+	LR–
SVM	Training	0.813 (0.745–0.882)	0.733	0.536	0.985	0.692	35.73	0.47
SVM	Test	0.765 (0.642–0.887)	0.672	0.417	1	0.588	∞	0.58
Neural network	Training	0.866 (0.809–0.922)	0.787	0.679	0.924	0.781	8.93	0.35
Neural network	Test	0.807 (0.695–0.918)	0.781	0.694	0.893	0.781	6.49	0.34
MLP	Training	0.836 (0.772–0.899)	0.773	0.631	0.955	0.757	14.02	0.39
MLP	Test	0.793 (0.679–0.907)	0.719	0.583	0.893	0.7	5.45	0.47
Gaussian process	Training	0.812 (0.743–0.881)	0.687	0.774	0.576	0.734	1.83	0.39
Gaussian process	Test	0.763 (0.642–0.884)	0.656	0.778	0.5	0.718	1.56	0.44
GBM	Training	0.997 (0.992–1.000)	0.98	0.976	0.985	0.982	65.07	0.02
GBM	Test	0.754 (0.633–0.875)	0.703	0.75	0.643	0.74	2.10	0.39
Logistic regression	Training	0.811 (0.742–0.879)	0.747	0.679	0.833	0.75	4.07	0.39
Logistic regression	Test	0.769 (0.649–0.889)	0.766	0.694	0.857	0.769	4.85	0.36
Naive Bayes	Training	0.82 (0.751–0.888)	0.76	0.583	0.985	0.731	38.87	0.42
Naive Bayes	Test	0.784 (0.669–0.898)	0.75	0.556	1	0.714	∞	0.44
XGBoost	Training	0.863 (0.806–0.920)	0.78	0.702	0.879	0.781	5.80	0.34
XGBoost	Test	0.766 (0.648–0.885)	0.734	0.722	0.75	0.754	2.89	0.37
Random forest	Training	1.000 (1.000–1.000)	1	1	1	1	∞	0.00
Random forest	Test	0.756 (0.633–0.880)	0.719	0.75	0.679	0.75	2.34	0.37

AUC, area under the curve; CI, confidence interval; GBM, gradient boosting machine; MLP, multilayer perceptron; SVM, support vector machine; XGBoost, extreme gradient boosting; LR+, positive likelihood ratio; LR–, negative likelihood ratio.

AUC quantifies the overall discriminative ability across all thresholds. Accuracy is the proportion of all correct predictions. Sensitivity measures the proportion of actual positives that are correctly identified. Specificity measures the proportion of actual negatives that are correctly identified. The F1-score represents the harmonic mean of precision and sensitivity, providing a balanced metric especially for imbalanced datasets.

The SHAP method was employed to provide a global interpretation of the prediction mechanism of the radiomics model (Neural Network). The SHAP importance bar plot revealed that among the three incorporated radiomics features, logarithm_glszm_ZoneEntropy made the highest contribution, followed by square_firstorder_Skewness and log.sigma.1.mm.3D_firstorder_Skewness (Figure 2K). The beeswarm (Figure 2L) and dependence plots (Figure 2M) demonstrated that decreased log.sigma.1.mm.3D_firstorder_Skewness values and increased logarithm_glszm_ZoneEntropy and square_firstorder_Skewness values were correlated with a higher risk of atherothrombosis. Local interpretation was performed on a high-risk patient (Sample 10), for whom the radiomics model predicted a disease risk of approximately 0.927. SHAP force (Figure 2N) and waterfall plots (Figure 2O) quantified the contributions of radiomics features, indicating that higher square_firstorder_Skewness made the largest positive contribution (SHAP = +0.207), followed by higher logarithm_glszm_ZoneEntropy (+0.0984) and lower log.sigma.1.mm.3D_firstorder_Skewness (+0.0608).

### 3.3 Clinical–radiomics fusion model development

**Figure 3:**
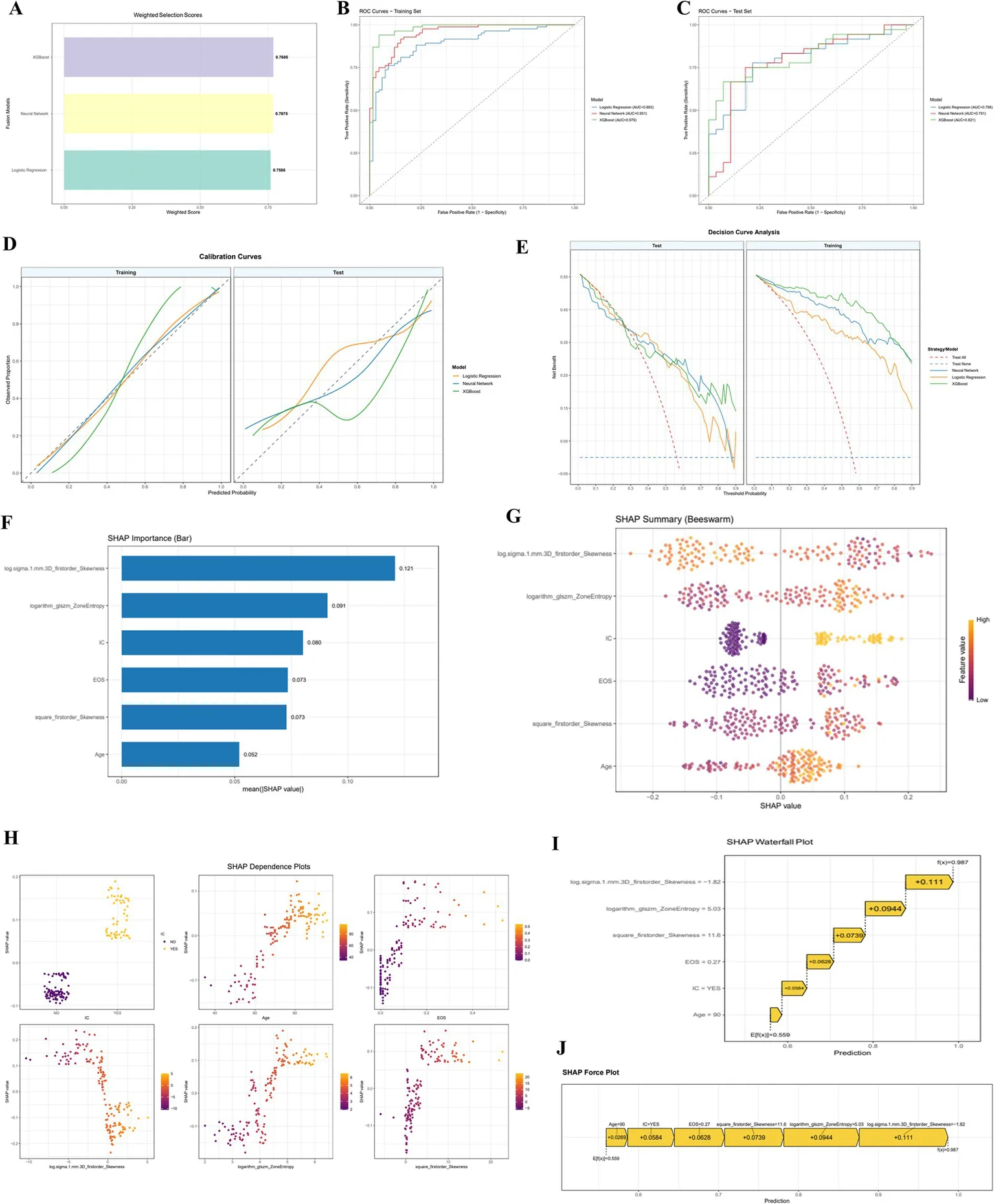
Performance evaluation and interpretability analysis of the clinical-radiomics fusion ML model. **(A)** Horizontal bar chart of the weighted performance score ranking for ML algorithms of the fusion model in the test set. **(B)** ROC curves of ML models in the training set. **(C)** ROC curves of ML models in the test set. **(D)** Calibration curves of the fusion model in the training and test sets. **(E)** Decision curve analysis of the fusion model in the training and test sets. **(F)** SHAP global importance bar plot of the fusion model. **(G)** SHAP summary beeswarm plot of the fusion model. **(H)** SHAP dependence plots of fusion variables. **(I)** SHAP waterfall plot for a typical high-risk sample (Sample 10). **(J)** SHAP force plot for a typical high-risk sample (Sample 10). ML, machine learning; ROC, receiver operating characteristic; SHAP, SHapley Additive exPlanations.

Comparative analysis of different ML algorithms in the testing sets of the clinical and radiomics models revealed that Neural Network, XGBoost, and Logistic Regression exhibited relatively superior performance across various evaluation metrics. Consequently, clinical-radiomics fusion prediction models were constructed using these three algorithms based on the selected clinical and radiomics features. Correlation analysis indicated that clinical features and radiomics features did not exhibit substantial correlation (Supplementary Figure S3E). Results showed that among the fusion models constructed using the three ML algorithms, XGBoost achieved the best performance with a weighted score of approximately 0.769 (Figure 3A). The AUCs of this model in the training and testing sets were 0.979 (95% CI 0.959–0.999) (Figure 3B) and 0.821 (95% CI 0.718–0.924) (Figure 3C), respectively. In the training set, the sensitivity, specificity, F1-score, and accuracy were 0.94, 0.939, 0.946, and 0.94, respectively. In the testing set, the corresponding metrics were 0.75, 0.714, 0.761, and 0.734. Additionally, the LR + and LR- were 2.62 and 0.35, respectively (Table 4).

**Table 4:** Performance metrics of the clinical-radiomics fusion model on the training and test sets.

Model	Dataset	AUC (95% CI)	Accuracy	Sensitivity	Specificity	F1	LR+	LR–
Neural network	Training	0.951 (0.921–0.981)	0.873	0.893	0.848	0.888	5.88	0.13
Neural network	Test	0.791 (0.672–0.909)	0.75	0.75	0.75	0.771	3.00	0.33
Logistic regression	Training	0.893 (0.841–0.945)	0.827	0.81	0.848	0.84	5.33	0.22
Logistic regression	Test	0.798 (0.688–0.907)	0.734	0.694	0.786	0.746	3.24	0.39
XGBoost	Training	0.979 (0.959–0.999)	0.94	0.94	0.939	0.946	15.41	0.06
XGBoost	Test	0.821 (0.718–0.924)	0.734	0.75	0.714	0.761	2.62	0.35

AUC, area under the curve; CI, confidence interval; XGBoost, extreme gradient boosting; LR+, positive likelihood ratio; LR-, negative likelihood ratio.

AUC quantifies the overall discriminative ability across all thresholds. Accuracy is the proportion of all correct predictions. Sensitivity measures the proportion of actual positives that are correctly identified. Specificity measures the proportion of actual negatives that are correctly identified. The F1-score represents the harmonic mean of precision and sensitivity, providing a balanced metric especially for imbalanced datasets.

Calibration curve analysis indicated that among the three models (Figure 3D), the XGBoost fusion model achieved the lowest Brier score (0.182) in the testing set (Supplementary Table S6), suggesting optimal calibration performance. DCA analysis indicated that the model provided clinical utility across most decision threshold ranges (Figure 3E). Collectively, the XGBoost fusion model exhibited excellent performance in terms of discriminative ability (AUC) and clinical decision utility. However, a notable difference in AUC was observed between the training and testing sets for the XGBoost fusion model. XGBoost is a powerful tree-based ensemble algorithm capable of capturing complex data patterns, which has a high capacity for fitting the training data, particularly when the training sample size is limited relative to the feature dimensionality. To further investigate this, learning curves were generated (Supplementary Figure S4). The learning curves showed that the performance on the independent test set exhibited an upward trend with increasing training samples and eventually stabilized, indicating that the model retains discriminative power on unseen data and may benefit from larger sample sizes. The SHAP method was employed to provide a global interpretation of the clinical-radiomics fusion model. The SHAP importance bar plot ranked the features in descending order of importance, specifically log.sigma.1.mm.3D_firstorder_Skewness, logarithm_glszm_ZoneEntropy, IC, EOS, square_firstorder_Skewness, and Age (Figure 3F). Furthermore, the SHAP beeswarm (Figure 3G) and dependence plots (Figure 3H) indicated that the directional associations between feature values and the predicted risk of atherothrombosis remained consistent with the influence patterns observed in the preceding clinical and radiomics individual models. Local interpretation was performed on Sample 10, where the waterfall (Figure 3I) and force plots (Figure 3J) displayed a predicted probability of 0.987. Lower log.sigma.1.mm.3D_firstorder_Skewness made the largest contribution to the model prediction (SHAP value = +0.111), followed by higher logarithm_glszm_ZoneEntropy (+0.0944), higher square_firstorder_Skewness (+0.0739), elevated EOS levels (+0.0628), the presence of IC (+0.0584), and advanced age (+0.0269).

Of note, the fusion model outperformed the simple clinical baseline model, the clinical model, and the individual radiomics models. This comparison indicates that the fusion model provides meaningful incremental diagnostic value.

## 4 Discussion

This study indicates that a clinical–radiomics fusion model has the potential to differentiate lower extremity arterial embolism from atherothrombosis in a single-center cohort (testing AUC = 0.821), suggesting that the integration of clinical and imaging features substantially enhances diagnostic discrimination.

In the predictive models, IC, advanced age, and EOS were identified as important clinical features for differentiating between lower extremity atherothrombosis and AE. Previous studies have confirmed that IC is a critical clinical indicator for differentiating between lower extremity atherothrombosis and acute AE (5, 25). From the perspective of clinical symptoms, compared with AE, lower extremity atherothrombosis typically occurs secondary to pre-existing atherosclerosis and is primarily caused by the rupture of unstable plaques (26). Given that intermittent claudication is one of the classic manifestations of chronic occlusive diseases in lower extremity arteries, particularly atherosclerosis (27), it holds substantial value in suggesting a thrombotic etiology. From a demographic perspective, patients with lower extremity atherothrombosis are generally older (28). Advanced age is a well-established risk factor for atherosclerotic diseases, with its pathological process often regarded as an aging-related degenerative change (29, 30). The increase in age is accompanied by a rising incidence of lower extremity atherosclerosis (31), which makes elderly patients presenting with acute limb ischemia more predisposed to an atherothrombotic etiology.

Finally, this study demonstrates that EOS has discriminatory value in differentiating lower extremity arterial embolism from atherothrombosis, with elevated EOS serving as a predictive marker for atherothrombotic etiology. Mechanistically, eosinophils have been implicated in the pathological processes of atherosclerosis and thrombosis, contributing to plaque instability and thrombus formation through inflammatory and procoagulant pathways (32–36). Previous studies have shown that eosinophils, as important effector cells of allergic inflammation, are closely associated with various atherosclerotic lesions and disease severity (37–41). Moreover, circulating eosinophil counts and the levels of their activation marker, eosinophil cationic protein (ECP), correlate positively with atherosclerosis severity (37, 42), and elevated eosinophil levels are associated with an increased risk of acute thrombotic events in coronary atherosclerotic disease (43–45).

Finally, the selected radiomics features may indirectly reflect the micro-pathological heterogeneity of thrombi from different etiologies. Zone Entropy is utilized to quantify the spatial complexity of image textures. Atherothrombosis typically occurs secondary to atherosclerotic plaque rupture, and its formation process is often accompanied by complex pathological changes, including lipid remnants, fibrous tissue, and inflammatory cell infiltration. Consequently, it may exhibit higher tissue heterogeneity, which translates to increased textural complexity on imaging. In contrast, embolic thrombi predominantly originate from cardiogenic emboli and are primarily composed of fibrin and erythrocytes. Because their tissue composition is relatively uniform, they tend to exhibit lower textural heterogeneity. Furthermore, Skewness reflects the asymmetry of the CT attenuation distribution within the lesions. Variations in thrombus composition and internal structure can affect the spatial distribution pattern of attenuation values, thereby driving changes in skewness-related features. In summary, these radiomics features may capture potential histological differences between thrombi of distinct etiologies. However, because no imaging-pathology correlation analysis was conducted in this study, the aforementioned explanations remain reasoned hypotheses based on current pathological knowledge. Future studies incorporating histopathological examinations of thrombectomy specimens are required to further validate the association between these features and thrombus composition, thereby enhancing the biological interpretability and clinical translation value of the model.

This study has several limitations. First, the single-center retrospective design may introduce selection bias and limit the generalizability of the findings. Crucially, the model has only undergone internal hold-out validation. Its performance may vary when applied to external populations using different CT scanners or acquisition protocols. Prospective, multicenter studies with independent external validation are essential before this model can be considered for clinical use. Second, although rigorous data imputation strategies were applied, residual bias cannot be entirely excluded, and the results should therefore be interpreted with caution. Additionally, the training sample size (*n* = 150) was modest relative to the initial feature pool (55 clinical features and 219 radiomics features), resulting in a suboptimal feature-to-sample ratio that may limit model stability and generalizability. Finally, the performance gap between training and testing sets suggests potential overfitting, which may be mitigated by larger sample sizes in future studies. Furthermore, the univariate comparisons presented in Table 1 were exploratory and uncorrected for multiple testing, which may increase the risk of type I errors; these findings should therefore be interpreted as exploratory.

## Data Availability

The original contributions presented in the study are included in the article/Supplementary material, further inquiries can be directed to the corresponding author.

## Glossary

AE: Arterial embolism
ALI: Acute limb ischemia
ALT: Alanine aminotransferase
APOA1: Apolipoprotein A1
APOB: Apolipoprotein B
AST: Aspartate aminotransferase
AUC: Area under the curve
AF: Atrial fibrillation
BASO: Basophil count
BASO-R: Basophil ratio
CAD: Coronary artery disease
CeVD: Cerebrovascular disease
CI: Confidence interval
CK-MB: Creatine kinase-MB
Crea: Creatinine
CTA: Computed tomography angiography
DBIL: Direct bilirubin
DCA: Decision curve analysis
DM: Diabetes mellitus
ECP: Eosinophil cationic protein
EOS: Eosinophil count
EOS-R: Eosinophil ratio
FAE: FeAture Explorer
GBM: Gradient boosting machine
GGT: Gamma-glutamyl transferase
GLCM: Gray-level co-occurrence matrix
GLDM: Gray-level dependence matrix
GLSZM: Gray-level size zone matrix
HBP: Hypertension
HDL-C: High-density lipoprotein cholesterol
hs-TNT: High-sensitivity troponin T
HU: Hounsfield unit
ICC: Intraclass correlation coefficient
IC: Intermittent claudication
INR: International normalized ratio
KNN: K-nearest neighbors algorithm
LBP: Local binary pattern
LASSO: Least absolute shrinkage and selection operator
LDL-C: Low-density lipoprotein cholesterol
ML: Machine learning
MLP: Multilayer perceptron
MONO-R: Monocyte ratio
MYO: Myoglobin
NEU: Neutrophil count
NEU-R: Neutrophil ratio
NGTDM: Neighboring gray tone difference matrix
NT-proBNP: N-terminal pro-B-type natriuretic peptide
PACS: Picture archiving and communication system
PCT: Plateletcrit
PLT: Platelet count
PT: Prothrombin time
PT-%: Prothrombin time activity
PT-INR: Prothrombin time-international normalized ratio
PT-RATIO: Prothrombin time ratio
ROI: Region of interest
SHAP: Shapley Additive exPlanations
SVM: Support vector machine
TBA: Total bile acid
TBIL: Total bilirubin
TC: Total cholesterol
TG: Triglycerides
TT: Thrombin time
XGBoost: Extreme gradient boosting
LR+: Positive likelihood ratio
LR-: Negative likelihood ratio
