# Supplementary material for: Development and internal evaluation of an interpretable machine learning model based on clinical and radiomics features to differentiate lower extremity arterial embolism from atherothrombosis
Source: Front Med (Lausanne). 2026 Aug 10;13:1827567. doi: 10.3389/fmed.2026.1827567 (PMC13502155; doi:10.3389/fmed.2026.1827567)
Supplement: Supplementary file 1 [file Data_Sheet_1.docx]

*Table S1 Comparison of baseline characteristics between the training and testing sets*

| **Characteristic** | **Unit** | **Training Set** (N=150)^1^ | **Test Set** (N=64)^1^ | | **P-Value**^2^ |
| --- | --- | --- | --- | --- | --- |
| **Group** | - |  |  | | 1.000 |
| Control |  | 66 (44%) | 28 (44%) | |  |
| Disease |  | 84 (56%) | 36 (56%) | |  |
| **Sex** | - |  |  | | 0.290 |
| Female |  | 48 (32%) | 26 (41%) | |  |
| Male |  | 102 (68%) | 38 (59%) | |  |
| **Age** | Years | 76.00 (64.00, 83.00) | 77.00 (69.50, 83.00) | | 0.373 |
| **DM** | - |  |  | | 0.904 |
| No |  | 112 (75%) | 49 (77%) | |  |
| Yes |  | 38 (25%) | 15 (23%) | |  |
| **HBP** | - |  |  | | 0.612 |
| No |  | 80 (53%) | 31 (48%) | |  |
| Yes |  | 70 (47%) | 33 (52%) | |  |
| **CeVD** | - |  |  | | 1.000 |
| No |  | 112 (75%) | 48 (75%) | |  |
| Yes |  | 38 (25%) | 16 (25%) | |  |
| **CAD** | - |  |  | | 0.989 |
| No |  | 126 (84%) | 53 (83%) | |  |
| Yes |  | 24 (16%) | 11 (17%) | |  |
| **AF** | - |  |  | | 0.154 |
| No |  | 117 (78%) | 56 (88%) | |  |
| Yes |  | 33 (22%) | 8 (13%) | |  |
| **IC** | - |  |  | | 0.798 |
| No |  | 94 (63%) | 42 (66%) | |  |
| Yes |  | 56 (37%) | 22 (34%) | |  |
| **Smoking** | - |  |  | | 0.583 |
| No |  | 98 (65%) | 45 (70%) | |  |
| Yes |  | 52 (35%) | 19 (30%) | |  |
| **Alcohol** | - |  |  | | 0.295 |
| No |  | 100 (67%) | 48 (75%) | |  |
| Yes |  | 50 (33%) | 16 (25%) | |  |
| **hs-TNT** | μg/L | 0.02 (0.01, 0.03) | 0.02 (0.01, 0.04) | | 0.296 |
| **MYO** | μg/L | 70.08 (33.00, 336.60) | 105.60 (35.91, 745.10) | | 0.249 |
| **CK-MB** | μg/L | 2.98 (1.69, 10.58) | 3.51 (2.02, 22.55) | | 0.109 |
| **NT-proBNP** | ng/L | 645.82 (222.60, 1,872.00) | 983.25 (348.43, 2,377.00) | | 0.121 |
| **TC** | mmol/L | 4.66 (3.92, 5.20) | 4.65 (3.95, 5.49) | | 0.738 |
| **TG** | mmol/L | 1.29 (1.01, 1.92) | 1.27 (1.01, 1.87) | | 0.734 |
| **HDL-C** | mmol/L | 1.31 (1.08, 1.63) | 1.32 (1.07, 1.60) | | 0.928 |
| **LDL-C** | mmol/L | 2.72 (2.13, 3.12) | 2.70 (2.17, 3.33) | | 0.752 |
| **APOA1** | g/L | 1.41±0.30 | 1.41±0.32 | | 0.913 |
| **APOB** | g/L | 0.92 (0.78, 1.05) | 0.91 (0.73, 1.13) | | 0.979 |
| **WBC** | ×10⁹/L | 9.44 (7.04, 12.83) | 9.60 (7.06, 12.76) | | 0.995 |
| **NEU** | ×10⁹/L | 7.66 (4.98, 10.51) | 7.45 (5.34, 10.71) | | 0.591 |
| **LYM** | ×10⁹/L | 1.13 (0.74, 1.62) | 1.10 (0.56, 1.59) | | 0.177 |
| **MONO** | ×10⁹/L | 0.55 (0.41, 0.72) | 0.53 (0.31, 0.73) | | 0.442 |
| **EOS** | ×10⁹/L | 0.05 (0.01, 0.12) | 0.02 (0.00, 0.13) | | 0.374 |
| **BASO** | ×10⁹/L | 0.03 (0.02, 0.04) | 0.02 (0.01, 0.03) | | 0.024 |
| **NEU-R** | % | 80.05 (71.00, 86.90) | 83.10 (73.10, 89.75) | | 0.125 |
| **LYM-R** | % | 12.70 (7.10, 19.30) | 11.35 (5.80, 17.50) | | 0.213 |
| **MONO-R** | % | 6.05 (4.40, 7.60) | 5.25 (3.80, 7.15) | | 0.131 |
| **EOS-R** | % | 0.50 (0.10, 1.70) | 0.20 (0.00, 1.50) | | 0.229 |
| **BASO-R** | % | 0.30 (0.20, 0.50) | 0.20 (0.10, 0.35) | | 0.032 |
| **RBC** | ×10¹²/L | 4.36±0.79 | 4.40±0.69 | | 0.728 |
| **HGB** | g/L | 134.45±25.95 | 134.23±21.24 | | 0.949 |
| **HCT** | - | 0.41 (0.37, 0.45) | 0.42 (0.37, 0.46) | | 0.953 |
| **MCV** | fL | 94.95 (90.00, 97.70) | 94.25 (90.80, 97.65) | | 0.573 |
| **MCH** | pg | 30.85 (29.50, 32.40) | 31.00 (29.60, 32.10) | | 0.960 |
| **MCHC** | g/L | 326.00 (320.00, 333.00) | 328.00 (319.50, 332.50) | | 0.634 |
| **RDW-SD** | fL | 44.65 (42.20, 48.20) | 44.15 (42.45, 46.35) | | 0.547 |
| **RDW-CV** | % | 13.70 (13.10, 14.50) | 13.60 (13.00, 14.60) | | 0.862 |
| **PLT** | ×10⁹/L | 204.00 (152.00, 286.00) | 187.00 (144.50, 252.00) | | 0.085 |
| **MPV** | fL | 9.85 (9.00, 11.00) | 9.95 (9.10, 10.80) | | 0.563 |
| **PCT** | - | 0.21 (0.16, 0.27) | 0.17 (0.15, 0.26) | | 0.067 |
| **PDW** | % | 16.20 (15.90, 16.50) | 16.25 (16.00, 16.60) | | 0.282 |
| **P-LCR** | % | 24.80 (19.30, 33.50) | 26.80 (21.25, 32.80) | | 0.363 |
| **ALT** | U/L | 21.85 (13.90, 36.20) | 23.65 (16.00, 41.20) | | 0.215 |
| **AST** | U/L | 25.75 (19.00, 37.20) | 31.40 (23.00, 53.90) | | 0.009 |
| **ALB** | g/L | 39.95 (37.70, 42.90) | 40.40 (36.95, 42.60) | | 0.771 |
| **GLO** | g/L | 27.55 (24.50, 31.40) | 29.85 (25.65, 33.05) | | 0.043 |
| **TBIL** | μmol/L | 11.55 (7.70, 16.90) | 12.80 (10.15, 17.30) | | 0.115 |
| **DBIL** | μmol/L | 4.40 (2.80, 7.00) | 4.45 (3.40, 7.00) | | 0.375 |
| **TBA** | μmol/L | 4.20 (2.60, 6.70) | 3.65 (2.50, 6.95) | | 0.744 |
| **GGT** | U/L | 34.30 (23.70, 57.80) | 38.15 (19.20, 62.60) | | 0.891 |
| **ALP** | U/L | 79.15 (64.30, 98.80) | 80.85 (66.95, 109.30) | | 0.183 |
| **PA** | mg/L | 191.30 (150.50, 236.20) | 173.75 (127.05, 215.80) | | 0.070 |
| **PT** | s | 13.35 (12.60, 14.30) | 13.65 (12.85, 14.40) | | 0.385 |
| **PT-INR** | INR | 1.02 (0.96, 1.10) | 1.05 (0.98, 1.12) | | 0.348 |
| **PT-RATIO** | - | 1.02 (0.97, 1.08) | 1.04 (0.98, 1.10) | | 0.331 |
| **PT-%** | % | 95.40 (85.00, 108.00) | 92.00 (84.00, 104.00) | | 0.376 |
| **APTT** | s | 33.60 (29.70, 38.90) | 34.60 (30.70, 39.15) | | 0.415 |
| **TT** | s | 17.50 (16.30, 18.90) | 17.65 (16.60, 19.15) | | 0.322 |
| **Fib** | g/L | 3.93 (3.20, 5.27) | 3.64 (2.97, 4.86) | | 0.215 |
| **Urea** | mmol/L | 6.49 (5.12, 9.07) | 6.79 (5.25, 9.48) | | 0.539 |
| **UA** | μmol/L | 338.60 (267.10, 434.10) | 319.45 (245.95, 406.35) | | 0.407 |
| **Crea** | μmol/L | 75.80 (59.80, 95.20) | 75.80 (61.35, 89.20) | | 0.934 |
| **GFR** | mL/min | 83.70 (60.50, 95.30) | 77.85 (62.45, 88.45) | | 0.269 |
| **Abbreviations:** AF, atrial fibrillation; ALB, albumin; ALP, alkaline phosphatase; ALT, alanine aminotransferase; APOA1, apolipoprotein A1; APOB, apolipoprotein B; APTT, activated partial thromboplastin time; AST, aspartate aminotransferase; BASO, basophil count; BASO-R, basophil ratio; CAD, coronary artery disease; CeVD, cerebrovascular disease; CK-MB, creatine kinase-MB; Crea, creatinine; DBIL, direct bilirubin; DM, diabetes mellitus; EOS, eosinophil count; EOS-R, eosinophil ratio; Fib, fibrinogen; GFR, glomerular filtration rate; GGT, gamma-glutamyl transferase; GLO, globulin; HBP, hypertension; HCT, hematocrit; HDL-C, high-density lipoprotein cholesterol; HGB, hemoglobin; hs-TNT, high-sensitivity troponin T; IC, intermittent claudication; LDL-C, low-density lipoprotein cholesterol; LYM, lymphocyte count; LYM-R, lymphocyte ratio; MCH, mean corpuscular hemoglobin; MCHC, mean corpuscular hemoglobin concentration; MCV, mean corpuscular volume; MONO, monocyte count; MONO-R, monocyte ratio; MPV, mean platelet volume; MYO, myoglobin; NEU, neutrophil count; NEU-R, neutrophil ratio; NT-proBNP, N-terminal pro-B-type natriuretic peptide; P-LCR, platelet-large cell ratio; PA, prealbumin; PCT, plateletcrit; PDW, platelet distribution width; PLT, platelet count; PT, prothrombin time; PT-%, prothrombin time activity; PT-INR, prothrombin time-international normalized ratio; PT-RATIO, prothrombin time ratio; RBC, red blood cell; RDW-CV, red cell distribution width-coefficient of variation; RDW-SD, red cell distribution width-standard deviation; TBA, total bile acid; TBIL, total bilirubin; TC, total cholesterol; TG, triglyceride; TT, thrombin time; UA, uric acid; WBC, white blood cell.  **Notes:** ^1^n (%); Median (Q1, Q3); Mean±SD; ^2^Continuous variables: normally distributed data are presented as mean±SD and compared using Welch t-test; non-normally distributed data are presented as median (IQR) and compared using Mann–Whitney U test. Categorical variables are presented as n (%) and compared using chi-square test. All P-values are rounded to three decimal places. | | | |  |  |

*Table S2 Calibration metrics of the clinical prediction model on the training and test sets*

| **Model** | **Dataset** | **Brier Score** | **Calibration Intercept** | **Calibration Slope** |
| --- | --- | --- | --- | --- |
| SVM | Training | 0.196 | 0.608 | 0.893 |
| SVM | Test | 0.229 | 0.462 | 0.624 |
| Neural Network | Training | 0.159 | -0.044 | 1.122 |
| Neural Network | Test | 0.216 | 0.218 | 0.587 |
| MLP | Training | 0.166 | -0.118 | 1.184 |
| MLP | Test | 0.214 | -0.042 | 0.763 |
| Gaussian Process | Training | 0.202 | -0.386 | 3.023 |
| Gaussian Process | Test | 0.220 | -0.230 | 2.068 |
| GBM | Training | 0.083 | -0.250 | 2.018 |
| GBM | Test | 0.282 | 0.228 | 0.227 |
| Logistic Regression | Training | 0.180 | 0.000 | 1.000 |
| Logistic Regression | Test | 0.214 | 0.028 | 0.681 |
| Naive Bayes | Training | 0.189 | 0.052 | 0.587 |
| Naive Bayes | Test | 0.235 | 0.015 | 0.360 |
| XGBoost | Training | 0.172 | -0.053 | 1.691 |
| XGBoost | Test | 0.208 | -0.015 | 1.159 |
| Random Forest | Training | 0.116 | -0.015 | 1.400 |
| Random Forest | Test | 0.222 | 0.133 | 0.516 |

**Abbreviations:** GBM, gradient boosting machine; MLP, multilayer perceptron; SVM, support vector machine; XGBoost, extreme gradient boosting.

**Notes:** The Brier score quantifies the accuracy of predicted probabilities; lower values indicate better calibration. The calibration intercept reflects the overall prediction bias (ideal value = 0). The calibration slope reflects the discrimination and fit of predicted values (ideal value = 1); a slope significantly < 1 suggests a risk of overfitting, while a slope > 1 indicates overly conservative predictions.

*Table S3 ICC of the three selected radiomics features*

| Radiomics Feature | ICC Value |
| --- | --- |
| log.sigma.1.mm.3D_firstorder_Skewness | 0.905 |
| logarithm_glszm_ZoneEntropy | 0.971 |
| square_firstorder_Skewness | 0.819 |

Abbreviations: ICC, intraclass correlation coefficient.

*Table S4 Bootstrap stability assessment of the three radiomics features*

| Feature | Selection Frequency |
| --- | --- |
| log.sigma.1.mm.3D_firstorder_Skewness | 0.922 |
| logarithm_glszm_ZoneEntropy | 0.544 |
| square_firstorder_Skewness | 0.465 |

Notes: Based on 1,000 bootstrap iterations on the training set, recording the proportion of iterations in which each feature was jointly selected.

*Table S5 Calibration metrics of the radiomics model on the training and test sets*

| **Model** | **Dataset** | **Brier Score** | **Calibration Intercept** | **Calibration Slope** |
| --- | --- | --- | --- | --- |
| SVM | Training | 0.201 | -1.486 | 5.937 |
| SVM | Test | 0.231 | -1.406 | 6.006 |
| Neural Network | Training | 0.142 | -3.169 | 6.855 |
| Neural Network | Test | 0.178 | -1.899 | 4.345 |
| MLP | Training | 0.156 | -2.359 | 5.971 |
| MLP | Test | 0.179 | -1.993 | 5.356 |
| Gaussian Process | Training | 0.199 | -9.381 | 17.960 |
| Gaussian Process | Test | 0.208 | -8.529 | 16.420 |
| GBM | Training | 0.030 | -6.719 | 15.057 |
| GBM | Test | 0.223 | -1.223 | 2.633 |
| Logistic Regression | Training | 0.164 | -2.720 | 5.738 |
| Logistic Regression | Test | 0.183 | -2.398 | 5.127 |
| Naive Bayes | Training | 0.190 | -1.228 | 5.042 |
| Naive Bayes | Test | 0.212 | -1.116 | 4.593 |
| XGBoost | Training | 0.152 | -4.351 | 9.189 |
| XGBoost | Test | 0.179 | -2.899 | 6.063 |
| Random Forest | Training | 0.025 | -75.633 | 151.767 |
| Random Forest | Test | 0.208 | -1.482 | 3.309 |

**Abbreviations:** GBM, gradient boosting machine; MLP, multilayer perceptron; SVM, support vector machine; XGBoost, extreme gradient boosting.

**Notes:** The Brier score quantifies the accuracy of predicted probabilities; lower values indicate better calibration. The calibration intercept reflects the overall prediction bias (ideal value = 0). The calibration slope reflects the discrimination and fit of predicted values (ideal value = 1); a slope significantly < 1 suggests a risk of overfitting, while a slope > 1 indicates overly conservative predictions.

*Table S6 Calibration metrics of the clinical-radiomics fusion model on the training and test sets*

| **Model** | **Dataset** | **Brier Score** | **Calibration Intercept** | | **Calibration Slope** |
| --- | --- | --- | --- | --- | --- |
| Neural Network | Training | 0.089 | -3.544 | 7.157 | |
| Neural Network | Test | 0.195 | -1.336 | 3.133 | |
| Logistic Regression | Training | 0.128 | -2.825 | 5.922 | |
| Logistic Regression | Test | 0.187 | -1.604 | 3.712 | |
| XGBoost | Training | 0.067 | -5.500 | 11.306 | |
| XGBoost | Test | 0.182 | -1.721 | 3.617 | |

**Abbreviations:** XGBoost, extreme gradient boosting.

**Notes:**The Brier score quantifies the accuracy of predicted probabilities; lower values indicate better calibration. The calibration intercept reflects the overall prediction bias (ideal value = 0). The calibration slope reflects the discrimination and fit of predicted values (ideal value = 1); a slope significantly < 1 suggests a risk of overfitting, while a slope > 1 indicates overly conservative predictions.

Figure S 1 Flowchart of patient selection.

Note: The diagram illustrates the criteria and process for patient inclusion and exclusion.

Figure S 2 ROC curves of the simple baseline model

Figure S 3 Quality control of data imputation and variable correlation analysis. (A) Density plots of clinical variables before and after KNN imputation, used to assess the consistency of data distribution in the training and testing sets. (B) Comparison of the distributions of original observed values versus imputed values for clinical variables in the training set, used to verify the reliability of the imputed data. (C) Comparison of the distributions of original observed values versus imputed values for clinical variables in the testing set. (D) Spearman correlation heatmap of clinical variables, used to identify and exclude variables with multicollinearity. (E) Correlation heatmap between clinical features and radiomics features used for modeling.

Figure S4 Learning curves for the XGBoost clinical‑radiomics fusion model
